# Nanosystems in Edible Coatings: A Novel Strategy for Food Preservation

**DOI:** 10.3390/ijms19030705

**Published:** 2018-03-01

**Authors:** María L. Zambrano-Zaragoza, Ricardo González-Reza, Néstor Mendoza-Muñoz, Verónica Miranda-Linares, Tania F. Bernal-Couoh, Susana Mendoza-Elvira, David Quintanar-Guerrero

**Affiliations:** 1 Laboratorio de Procesos de Transformacion y Tecnologías Emergentes de Alimentos, FES-Cuatitlan, Universidad Nacional Autonoma de Mexico, Cuautitlán Izcalli 54714, Mexico; gonzalez.reza@comunidad.unam.mx (R.G.-R.); veronica.miranda.linares@comunidad.unam.mx (V.M.-L.); bernalita.couoh93@gmail.com (T.F.B.-C.); 2 Laboratorio de Farmacia, Facultad de Ciencias Químicas, Universidad de Colima, Coquimatlan 28400, Mexico; nmendoza0@ucol.mx; 3 Laboratorio de Microbiología y Virología de las Enfermedades Respiratorias del Cerdo, FES-Cuatitlan, Universidad Nacional Autonoma de Mexico, Cuautitlán Izcalli 54740, Mexico; seme@unam.mx; 4 Laboratorio de Posgrado en Tecnología Farmacéutica, FES-Cuatitlan, Universidad Nacional Autonoma de Mexico, Cuautitlán Izcalli 54740, Mexico

**Keywords:** nanostructures, active compounds, essential oils, biopolymers

## Abstract

Currently, nanotechnology represents an important tool and an efficient option for extending the shelf life of foods. Reducing particle size to nanometric scale gives materials distinct and improved properties compared to larger systems. For food applications, this technology allows the incorporation of hydrophilic and lipophilic substances with antimicrobial and antioxidant properties that can be released during storage periods to increase the shelf life of diverse products, including whole and fresh-cut fruits and vegetables, nuts, seeds, and cheese, among others. Edible coatings are usually prepared with natural polymers that are non-toxic, economical, and readily available. Nanosystems, in contrast, may also be prepared with biodegradable synthetic polymers, and liquid and solid lipids at room temperature. In this review, recent developments in the use of such nanosystems as nanoparticles, nanotubes, nanocomposites, and nanoemulsions, are discussed critically. The use of polymers as the support matrix for nanodispersions to form edible coatings for food preservation is also analyzed, but the central purpose of the article is to describe available information on nanosystems and their use in different food substrates to help formulators in their work.

## 1. Introduction

Global trends in consumption show a preference for natural products, especially those of vegetable origin. Consumer demand clearly favors minimally-processed products to avoid causing substantial changes in their fresh characteristics, and maintain the nutritional and functional properties of different components of fruits, vegetables, seeds, and nuts, etc. In this regard, edible coatings—a thin layer formed on the food surface to extend its shelf life—represent one of the best ways to preserve the properties, functionality, and characteristics of foods at a low cost. These coatings are easy to apply by spraying, immersion, or rubbing, and are prepared with environmentally-friendly materials. Finally, they rarely need to be eliminated before consumption [1]. Recently, these applications have also been considered an effective method of vectorizing natural ingredients with antimicrobial and antioxidant activity to maximize their beneficial effects on the quality of fresh produce [2,3]. These uses have been welcomed by consumers because, when used in conjunction with refrigerated storage, they increase both the safety and quality of food products, and because they are prepared with natural ingredients, such as proteins, polysaccharides, essential oils (EOs), and other organic substances and/or inorganic compounds, all of which help increase the benefits of the products so treated [4,5].

The properties and functionality required for edible coatings must be developed mainly in relation to the deterioration pathways of each food product, so they depend on the composition of individual foods; for example, it may be necessary to decrease oxygen permeability to protect products that are sensitive to oxidation, or reduce respiration rates and the production of ethylene in fruits, or prevent weight loss. In addition, edible coatings must be compatible—both organoleptically and functionally—with the specific food involved [6]. In this regard, the use of EOs and other extracts of natural origin has been evaluated in the development of edible coatings, given their demonstrated potential to inhibit the microbial growth that damages products and decreases shelf life. These coatings can also utilize the antioxidant capacity of extracts and EOs to scavenge free radicals, and so decrease deterioration rates and prevent oxidation reactions caused by interaction with components of the foods.

Today, nanotechnology represents an area of opportunity for developing vehicles to transport certain EOs, vitamins, and other plant extracts, such as polyphenols, with antimicrobial and antioxidant properties. Also, their submicron size offers a new way to modify gas transport properties and the release of natural products, while improving mechanical resistance, transparency, functionality, and antioxidant and antimicrobial activity. Nanotechnology includes all submicron-size systems (<1000 nm), preferably those measuring 100–500 nm [7]. Their greater surface area per mass compared to larger particles of the same chemical composition make nanosystems more stable and biologically-active, while permitting the incorporation of hydrophobic and/or active substances that do not greatly modify their appearance or transparency, but maintain the visual characteristics and help increase the shelf life of foods [8].

Recently, applications of this emerging technology to the development of edible coatings have included various nanosystems, including polymeric nanoparticles, nanoemulsions and nanocomposites, among others, in efforts to control the release of EOs, polyphenols, and fat-soluble vitamins, compared to their function in microcapsules and other forms of incorporation into polymeric matrices. These include EOs of oregano and thyme, whose active agents are thymol and carvacrol, respectively, two substances with good antimicrobial effect. Another aspect considered is antioxidant capacity, which reduces the rate of reactions and enzymatic activity of, for example, polyphenol oxidases, peroxidases, and lipoxygenases. The use of nanosystems has thus propitiated exploration of ways to control the release of elements into the environment, by acting as a barrier in conjunction with the polymer matrix into which they are incorporated [9,10,11]. Nanosystems have distinct morphologies that modify the characteristics of edible coatings in different ways [12]. It is noteworthy that regulation of the use of nanosystems in foods is a controversial issue. In the United States, Regulation 258/97 establishes that if a nanomaterial is used as a primary ingredient, then it needs to be considered as a “Novel Food” [13]. When the term “food additive” is employed (1333/2008), even if its composition is authorized as raw material, it is not necessarily applicable to the nano form, and the nanosystem requires consideration as a different additive; thus, it needs to be evaluated as a new, safe material before it can be placed on the market.

The main aim of this paper was to review the use of nanosystems as polymeric-support matrices to form edible coatings for food preservation. Also, we focused on describing available information on nanotechnological applications and their use in different food types, to aid formulators in their work.

## 2. Nanosystems as Components of Edible Coatings

Advances in the preparation of nanosystems that incorporate ingredients acceptable for food products have made it possible to explore the functional modifications of edible coatings that integrate nanoemulsions, polymeric nanoparticles, nanofibers, solid lipid nanoparticles, nanostructured lipid carriers, nanotubes, nanocrystals, nanofibers, or mixtures of organic and inorganic nano-sized components. Generally, these nanosystems are incorporated into polysaccharide or protein matrices called “nanocomposites”, which are defined as the combination of two or more materials, to form a mixture that improves the properties of a component in which at least one of them has nanometric scale [14]. The development of nanocomposites has allowed edible coatings to be used as “temporal distribution systems” that release active substances from a matricial film to the food, to improve conservation [15].

### 2.1. Nanoemulsion

Nanoemulsions are colloidal systems that consist of an oil phase dispersed in an aqueous phase, such that each drop of oil is surrounded by a thin interfacial layer made up of emulsifying molecules [16]. The particle size of nanoemulsions ranges from 50–500 nm [12]. Two kinds of nanoemulsions are available, depending on the phases: oil/water (o/w) or water/oil (w/o). However, o/w systems are preferred for producing edible coatings, since they allow the incorporation of different lipophilic substances with antioxidant and antimicrobial effects into a hydrophilic polymeric matrix.

Nanoemulsions are thermodynamically unstable but kinetically stable. However, time-dependent changes can cause different destabilization effects and result in aggregation, coalescence, flocculation, Oswald’s maturation, and gravitational sedimentation [17,18]. The stability of nanoemulsions is a function of particle size and monodispersity, which implies that they can be diluted without changing the size distribution of the drops. Nanoemulsions are highly stable in relation to gravitational separation, due to their relatively small particle size, which allows the effects of Brownian movements to dominate gravitational forces [12,19].

The lipid phase of nanoemulsions may consist entirely of the bioactive lipids (e.g., essential oil, oily flavor, oily color, vitamin, etc.), or of oily solutions in which lipophilic components are first dissolved in an oil (corn, soybean, sunflower, olive, etc.). Examples of lipophilic compounds in nanoemulsions are bioactive, flavoring, antimicrobial, antioxidant, and nutraceutical lipids [19]. A potential advantage of reducing the size of the oil drops to nanometer range is that it increases the solubility of the bioactive lipids in the surrounding water phase, which enhances bioactivity, desirability and palatability [20,21].

The lipophilic materials that can be incorporated into nanoemulsions include essential oils from plants (e.g., oregano, sage, clove, mint, limonene), fatty acids (ω-3 fatty acids, conjugated linoleic acid, butyric acid), carotenoids (β-carotene, lycopene, lutein, zeaxanthin), antioxidants (tocopherols, flavonoids, polyphenols), phytosterols (stigmasterol, β-sitosterol, campesterol) and quinones (coenzyme Q_10_) [22].

The use of nanoemulsions in the preparation of edible coatings began to be explored recently because their characteristics, and inclusion in a polymeric matrix allows greater control over the release of active ingredients.

### 2.2. Polymeric Nanoparticles 

Polymeric nanoparticles are colloidal structures that measure 100–1000 nm. Two types of polymeric nanoparticles can be described in terms of morphology and architecture: nanospheres and nanocapsules. Nanospheres are formed by a dense polymeric matrix, while nanocapsules are composed of an oil core surrounded by a polymeric membrane. In the case of nanospheres, the target (bioactive) molecule to be trapped can be adsorbed into the surface or dispersed molecularly in the matrix. With nanocapsules, in contrast, the bioactive ingredient can be retained in an oily core surrounded by a thin polymeric wall [1]. Although polymeric nanoparticles can be more common dissolved in the oil core. Despite that polymeric nanoparticles could be prepared by monomers, this technique is usually avoided due to the presence of residual toxic compounds (e.g., monomers, oligomers, initiators) after the polymerization reaction [23]. Therefore, methods that use preformed polymers are preferred. In both systems—nanospheres and nanocapsules—the kind of polymer utilized has a key role in the most significant characteristics of the polymeric nanoparticles related to the active compound; for example, in the entrapment/encapsulation efficiency of the active compound, the release rate, the degradation process, and the protective ability, to name just a few. Common examples of preformed biodegradable polymers used to prepare polymeric nanoparticles for food applications are poly-ε-caprolactone (PCL), polylactic acid (PLA), poly-d,l-lactide-*co*-glycolide (PLGA), cellulose acetate phthalate (CAP), ethylcellulose, alginate, and chitosan [24].

Though the development of polymeric nanoparticles was first reported in the pharmaceutical field for drug delivery systems, less than two decades ago, these systems captured the interest of the food sector as emerging applications that could provide innovative solutions to such issues as protecting nutraceuticals from degradation, the delivery and controlled release of nutraceuticals across edible coatings, taste-masking, and active or intelligent packaging.

It is possible to produce active edible coatings when antioxidants or antimicrobial molecules are encapsulated into polymeric nanoparticles. In these formulations, the compound may be delivered or have its delivery prolonged or controlled, to create an on-demand microenvironment that improves the shelf life of foods. Edible coatings are packing systems that are highly-predisposed to incorporate polymeric nanoparticles, because of the chemical compatibility of the nanoparticle matrix, or shell, and the composition of the edible coating support. This ensures that the compounds will be well-dispersed over the surface of the treated food [25].

Several studies of the encapsulation of antioxidant or antimicrobial compounds derived from natural sources into nanocapsules or nanospheres have been reported for food and pharmaceutical applications, but the utilization of these systems in edible coatings has not yet been extensively documented. Polyphenols are examples of antioxidant actives that have been successfully incorporated into nanocapsules and have the potential to be used in edible coatings. One of the most widely-studied polyphenols is curcumin, an extract of the rhizome of turmeric whose biological activity has been amply-described. Its antioxidant and antimicrobial properties are two of the most useful attributes for food applications. Several nano-formulations that include curcumin in nanospheres or nanocapsules have been reported in the scientific literature; for example, Liu et al. (2017) [26] described the preparation of nanocapsules with curcumin by means of the one-pot method for the inhibition of Millard reaction in milk model. Liu et al. [27] also reported elaborating curcumin nanospheres using chitosan as the polymer, while Lv et al. (2014) [28] reported the incorporation of jasmin essential oil nanocapsules composed of two biocompatible polymers—gelatin and arabic gum—by complex coacervation. In yet another approach, Coradini et al. [29] reported the co-encapsulation of resveratrol and curcumin in PCL/grape seed oil (polymer/oil) nanocapsules. Polyphenols, such as epigallocatechin gallate [30], quercetin [24,31,32,33], and hydroxycinnamic acids [34], have also been incorporated into polymeric nanoparticles with potential for use in edible coatings. Other antioxidant molecules, like the carotenoids, have been successfully included in nanocapsules, for example, Queiroz et al. [35] incorporated lycopene-PCL nanocapsules into cassava starch films to evaluate them as biodegradable coatings. In another works, plant extracts such as peppermint oil were employed due to their antimicrobial effect [36], and lutein was encapsulated into PCL nanocapsules by the interfacial deposition technique [37]. Finally, β-carotene incorporated into PCL nanocapsules by the emulsification-diffusion method also showed potential for use in food technology [38].

### 2.3. Solid Lipid Nanoparticles

The use of solid lipid nanoparticles (SLNs) for the encapsulation and delivery of bioactive components dates from 1990, when these nanostructures were manufactured by replacing the liquid state lipid (oil) in the emulsion with a solid one, such that the lipids are solid at both ambient and body temperature [39]. More recently, SLNs have attracted increasing attention as a colloidal carrier system for biologically-active food components [40], and as an alternative to traditional colloidal carriers [41,42], due to certain unique properties; namely, small particle size, large specific surface area, solidity, particle shape, and surface chemistry. This has generated enormous enthusiasm and anticipation for potential applications [43].

This system consists of spherical solid lipid particles in the submicron range of 50–1000 nm [40,41,44] into which the water-insoluble core is dispersed and then stabilized by surfactants [45]. The solid core contains the bioactive compound, either dissolved or dispersed in the solid high-melting-point oil matrix, or the fatty acid chains [41], which during transition to more stable polymorphic forms—from α to β′ to β—can physically displace the molecules from the crystalline lattice [39]. The use of solid lipids in the dispersed phase allows the controlled release of the encapsulated compounds, as the degradation of the lipid structure gradually releases the lipophilic or hydrophilic bioactive compounds [39,41]. SLNs are typically prepared using the hot homogenization process, in which a lipid and an aqueous surfactant solution are homogenized at a temperature above the lipid’s melting point to produce an oil–water nanoemulsion [46].

### 2.4. Lipid Nanocarriers

Lipid-based nanocarriers have been widely described during the last decade as ideal systems for the transport and delivery of active substances that need to be protected from enzymatic and biological degradation [47,48,49], and as an option for resolving the common solubility problems that affect lipophilic substances [8,50,51]. A mix of solid and liquid lipids is used to formulate nanostructured lipid carriers (NLCs), usually in a ratio of 70:30 [49].

Nanostructured lipid carriers (NLCs) are a second generation of lipid-based nanoparticles, developed by Müller in 2000 to overcome problems with SLNs, due to the process of the full crystallization of fat that reduces drug solubility and causes expulsion of the active substances from the lipid particles [52]. They are derived from oil-in-water nanoemulsions in which the inner phase consists of a solution of the bioactive compound in a lipid matrix formed by a mixture of solid and liquid lipids (oils), while the outer phase is usually water mixed with emulsifiers. NLCs are specially-designed to encapsulate hydrophobic compounds, since introducing a liquid lipid allows a better dispersion of the active substances and causes a decrease of the melting point compared to a pure solid lipid, and of defects in the crystal structure that prevent lipid polymorphism and avoid a perfect crystalline structure to provide more space for the active components. This also inhibits expulsion of the active substances to achieve greater drug loading and stability [45,48,49,53,54,55,56]. Other advantages of NLCs are that they present greater colloidal stability, due to the higher density of the solid lipid, they eliminate the need to use organic solvents during production, and they are less prone to changes in particle shape. Also, their easy dispersion into an aqueous medium, nanoscale size, and biocompatibility enhance their ability to deliver nutraceuticals to the body [56,57]. The lipid core of NLCs is composed primarily of medium-chain triglycerides that encapsulate the lipophilic components, surrounded by the solid lipid; while the outer surface is stabilized using surfactants, such as lecithin [48].

Most applications of NLCs were introduced in pharmaceutical science, so little research is available on their applications in the food field [57]. One of the main challenges for using NLCs in food is that this requires a proper selection of food-grade ingredients, due to the chronic, long-lasting use of food products. This makes formulations difficult, because it significantly reduces the number of usable ingredients.

Lipids are the main ingredients of lipid nanoparticles that affect the properties of systems, including particle size, stability, the loading capacity of the active substance, and the sustained release behavior of the formulations. Increasing the solid/liquid ratio reduces particle size, while the concentration of the stabilizer and the ratio of active substance/lipid affects entrapment efficiency [45,49,58]. The lipids selected to entrap active substances must have certain well-defined characteristics, such as biocompatibility, good solubility of lipophilic ingredients in the lipid matrix, high stability against decomposition factors, high biodegradability, and innocuousness [45,51]. Moreover, the solid lipids must have a melting point above room temperature, while because the melting point of liquid lipids is much lower than ambient and body temperature, it is necessary to use surfactants to achieve fine dispersions of lipids in aqueous media [45,57,58].

Recently, many researchers in the food industry have been working to encapsulate active substances, mostly lipophilic ones, via NLCs. The main objective in most works is to encapsulate substances obtained from natural sources that exhibit one or more beneficial properties (antioxidant, antimicrobial, nutraceutical, colorant, etc.). Similarly, there is an increasing tendency to use solid and liquid lipids found in nature that can function to encapsulate active substances and, at the same time, provide some benefit to either the food products or directly to consumer health. For example, Huang et al. [59] developed NLCs to encapsulate quercetin using glyceryl monostearate as the solid lipid, linseed oil as the liquid lipid, and a mixture of Tween^®^ 80 and polyglycerol monostearate as the surfactant. Quercetin has been studied because of its therapeutic effects, which include antioxidant, anticancer, antibacterial, and anti-inflammatory actions, and its potential ability to prevent neurodegenerative diseases. Linseed oil, meanwhile, is known to be a good replacement for fish oil because of its high α-linoleic acid content, which is used to prevent cardiovascular diseases, hypertension, and inflammation, while also promoting brain function and antioxidant properties.

Other lipids derived from natural sources used in NLC production are cocoa butter, beeswax, carnauba wax, oleic acid, and soybean, grape seed, sunflower, and corn oil. Some of the natural hydrophilic substances encapsulated are vitamins, berry oils, curcumin, green tea extract, and carotenoids like β-carotene and lycopene [45,52].

### 2.5. Inorganic/Organic Nanocomposites in Edible Films

Due to their origin and properties, proteins, lipids, and polysaccharides have been used in the preparation of edible coatings. These components have also been prepared by mixing different proportions of each one to improve their barrier properties to gases, since hydrocolloids are characterized by providing a good barrier to oxygen, but a poor one to water vapor, though this can be enhanced by adding a lipid substance [11,60]. At present, due to the demand of minimally-processed—“healthful”—products, there is a constant challenge to develop new and more efficient edible coatings. A very attractive strategy is to elaborate coatings that incorporate nanosystems mixed with organic and inorganic substances that produce nanocomposites. These mixtures represent a recent option for improving the properties of edible coatings, since they permit better mechanical resistance, transparency, controlled release, and more effective gas barrier properties [11,60]. The most widely-used inorganic components for modifying the properties of edible coatings include montmorillonite (MMT), nano-SiOx, nano-TiO_2_, and nano-ZnO, as well as silver nanoparticles, though it is important to note that the latter can only be used to coat whole fruits and vegetables [61]. The following section describes the properties of the materials that are allowed as food additives and that have been prepared in nanometric sizes. Studies have shown that these systems function as modifiers of mechanical resistance and the release of active ingredients in edible coatings. Zinc oxide nanoparticles (Nano-ZnO)—one type of multifunctional inorganic nanoparticles—are known to inhibit microbial growth. Since zinc oxide has strong antimicrobial effects, it has been listed as a GRAS (Generally Recognized as safe) by the US FDA. Zinc has been used to fortify many products, and its use as a food additive is permitted. Nanotechnology provides opportunities to create new products with a wide range of applications, including the development of edible coatings with antimicrobial properties [62].

Nano-SiO_2−x_ belongs to a group of amorphous substances with a three-dimensional network structure. Its derivates have a stable silicone–oxygen structure because of the lack of oxygen on their surfaces. Its molecular formula is SiO_2−x_, with values of *x* from 0.4 to 0.8. Due to their small size, large specific surface area, high surface energy, unsaturated chemical bonds, and hydroxyl groups on the surface, nano SiOx are easily dispersed among macromolecular chains, as has been reported for modified starches [63], where they were found to positively modify the mechanical properties of biodegradable starch/nano-SiO_2_/PVA films. Nano-SiO_2_ is widely used in food products, and is registered in CODEX-Alimentarius as a food additive (E551). It is mainly used to thicken pastes, as an anti-caking agent to maintain flow properties in powdered products, and as a carrier for fragrances or flavors in food and non-food products [64].

Nanoclay is an abundant, natural nanoparticle substance that can be transformed into reinforcing agents. Nanoclays, such as montmorillonite (MMT), contain silicate, and have a multi-layered shape with a width and length of 100–150 nm, but its thickness (only 1 nm) is the crucial factor in improving the mechanical properties of composite films [65].

In addition to considering the characteristics of the inorganic material involved, it is important to establish how it will be incorporated into the polymer matrix, since this depends on whether its inorganic structure is intercalated with, incorporated into, or surrounded by, the polymer. This will affect its functionality and modify mechanical, optical, and transport properties in relation to the substance to be encapsulated. All materials used in food preservation must be safe and compatible with the food, so that they can help improve mechanical properties, thermal resistance and the controlled release of active compounds.

### 2.6. Nanotubes and Nanofibers

In the search for new ways to improve controlled release and to take advantage of active ingredients to protect fresh foods and/or provide them with specific applications, such as to carry substances with a positive effect during food storage and functionality at consumption time, including their use in edible coatings, nanotubes and nanofibers have recently been considered potentially useful carriers of active substances (antioxidants or antimicrobials) [10]. Carbon nanotubes have been most widely studied, but they are used mainly in the development of containers, since they have the capacity to be incorporated into polymeric matrixes (polysaccharides, proteins, and/or crystalline materials, such as solid lipids) with the aim of modifying mechanical properties, especially tensile strength and elasticity. For food applications, recent studies have considered nanotubes prepared with milk proteins, especially nanotubes of α-lactalbumin by partial hydrolysis. These nanotubes have cavities 8 nm in diameter that can encapsulate different active materials in foods. When these compounds are prepared with milk proteins for use in food preservation, there are few limitations on their incorporation into edible coatings, though it is important to consider the effect of humidity on their stability. Like the material to be encapsulated, they usually remain on the surface of the nanotube, thus achieving a controlled release through the edible coatings, depending on the support polymer used [66].

Nanofibers are another type of nanosystem. Their potential use has been explored in relation to the encapsulation of antioxidants and antimicrobial substances to preserve food quality and safety. They can be used as components of edible coatings. Nanofibers are known as fibrous scaffolds of nanometric size that have diameters less than 100 nm or even 500 nm. They have been used to immobilize enzymes, modify film properties, and encapsulate various active ingredients. In this sense, they are seen as a novel alternative for the development of edible coatings [67].

## 3. Nanosystems in Edible Coatings

Particles of submicron size show diverse possibilities for incorporation into food-grade polymeric matrixes that serve as support systems, while nanosystems help modify the properties of edible coatings. Therefore, different forms of systems contribute differently upon interacting with the coating matrix. The main modifications that have been reported relate to mechanical and optical properties, antioxidant and antimicrobial effects, and the possibility to achieve controlled release during storage of food products at different temperatures, especially in the cold storage of minimally-processed products [68,69]. These properties are useful for resolving food safety problems related to the growth of pathogenic microorganisms, because they can reduce the development of yeast, mold, and bacteria that deteriorate food during storage, thus decreasing their shelf life. The functionality of nanosystems serves to incorporate antioxidants, making it possible to reduce deterioration rates. Highlighting that today there are many natural antioxidants, for example, those obtained from essential oils that require nanoencapsulation that allow their use in the preparation of edible coatings that allow these to be carried through hydrophilic polymer matrices [70,71,72]. This is feasible with nanosystems, because they allow the use of smaller proportions of such substances, and so do not transfer flavor changes to the food; yet another positive effect. The next section presents a brief analysis of the applications of different nanosystems, some of which have already been incorporated into polymeric matrices to form edible coatings, while others are being explored for their potential to encapsulate antioxidant and antimicrobial substances.

### 3.1. Nanoemulsions in Edible Coatings

Nanoemulsions have considerable potential as delivery systems for active compounds in edible coatings and other applications in food-processing. A series of specialized studies have highlighted the potential advantages of using nanoemulsions in food. Table 1 shows a list of functional lipophilic compounds that have been nanostructured in nanoemulsion systems, showing their functionalities and fields of application.

**Figure 1:**
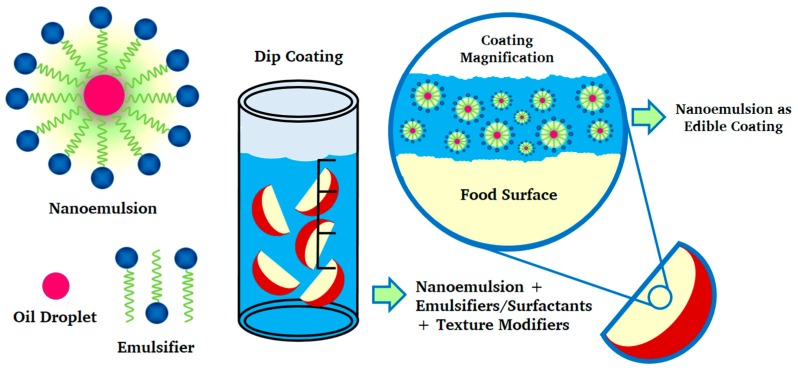
Nanoemulsion in edible coatings, food interaction.

Studies of the incorporation of nanoemulsions with bioactive compounds in food are numerous, but current commercial applications are limited. Figure 1 shows the interaction of foods with nanoemulsions formed in edible coatings with a polymeric matrix. Although research has shown that the nanostructuring of bioactive lipophilic ingredients in nanoemulsions increases their bioavailability, their real benefits after incorporation into complex food matrices and after food-handling operations have not yet been confirmed [22].

### 3.2. Polymeric Nanoparticles in Edible Coatings

Other compounds with high potential for inclusion in polymeric nanoparticles followed by incorporation into edible coatings are essential oils (EOs), since their antimicrobial activity has been recognized for many years. Alginate–chitosan nanocapsules of turmeric oil and lemongrass oil were reported by Natrajan et al. [81], who prepared nanoparticles by the pre-gelation of an oil in a water-based nanoemulsion of alginate by adding a calcium chloride solution. Finally, chitosan was added to crosslink the preformed alginate nanocapsules. Lemon grass oil has also been incorporated into cellulose–acetate nanocapsules by the nanoprecipitation method [82]. In another case, tragacanth gum—a natural material recognized as safe—was used to prepare peppermint oil nanocapsules by the microemulsion method [36]. Although these nanocapsules have been applied in the pharmaceutical field, few reports on the use of EO nanocapsules in food technology have been published, but Mohammadi et al. [83] recently described the encapsulation of *Zataria multiflora* essential oil in chitosan nanoparticles to prepare a coating that improves antioxidant activity and extends the shelf life of cucumbers.

**Figure 2:**
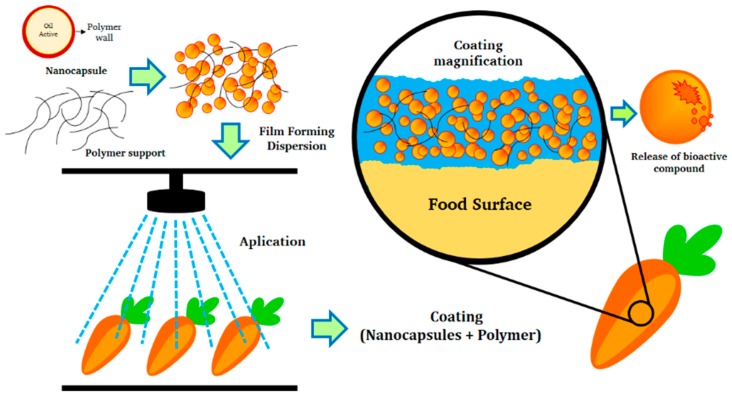
Structure of polymeric nanoparticles in edible coating.

Although several preformed polymers are available for the synthesis of polymeric nanoparticles, special attention has been paid to preparing nanoparticles with chitosan for food applications. Chitosan is non-toxic, biocompatible, biodegradable, possesses good mechanical properties and film-forming ability, and has selective permeability to gases, as well as fungicidal and antimicrobial properties. Edible coatings based on a solution of chitosan have been reported for decades [36,72,78,84,85,86,87,88], but few studies have reported using chitosan nanoparticles, though Mustafa et al. [88] described including them in an edible coating to preserve post-harvest tomatoes. In their study, the delay in color evolution in the coated fruits and the maintenance of quality during storage were the main benefits of the nanoparticles. However, that coating had low adhesion and durability, as shown by the breakdown of its moisture barrier properties, which meant that this edible coating required a support matrix. Pilon et al. [89], meanwhile, found differences between the conventional chitosan coating process (dipping into chitosan gel) and nanoparticle coating: when the nanoparticles were sprayed on fresh-cut apples, they formed a non-continuous coating that compromised the moisture barrier, though they provided a well-dispersed coating that had a greater antimicrobial effect on microorganisms, due to greater surface interaction. Eshghi et al. [90] described applying chitosan nanoparticles prepared by ionotropic gelation on fresh strawberries. In this case, the nanoparticles provided an effective control in reducing weight loss and maintaining firmness, while also delaying changes in the respiration rate for 3 weeks. Recently, Martínez-Hernández [91] reported the encapsulation of carvacrol, a major component of the essential oil of oregano, thyme, marjoram, and summer savory into polymeric chitosan nanoparticles prepared by ionotropic gelation. Those nanoparticles were used to protect fresh-cut carrots. Chitosan-based edible coatings are more accessible in terms of cost than other polymers, such as PLA or PCL. Figure 2 is a representation of nanoparticle distribution (nanospheres or nanocapsules) containing active components (essential oils or others with antioxidant and antimicrobial activity) into a polymeric matrix on a food surface. Release can be performed by diffusion in both cases (nanospheres or nanocapsules) or by membrane breaking in the case of nanocapsules.

### 3.3. Solid Lipid Nanoparticles (SLNs)

**Figure 3:**
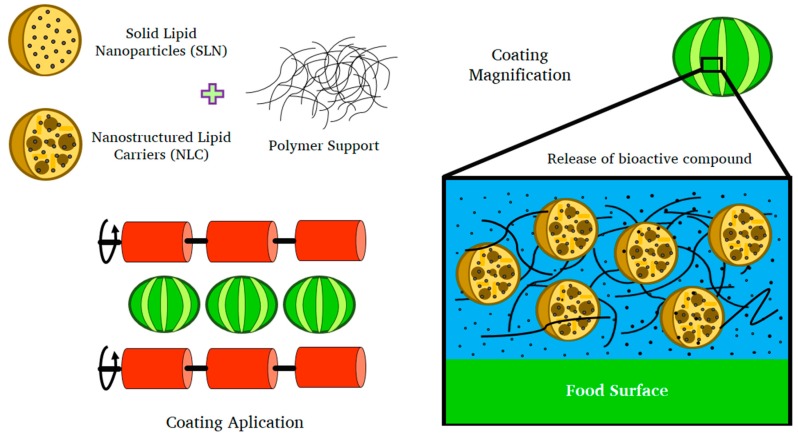
Solid lipid nanoparticles and nanostructured lipid carrier in edible coatings.

Solid lipid nanoparticles represent a promising system for the delivery of bioactive components in functional foods and as a component of edible coatings, due to their remarkable stability and high loading capacity. Figure 3 shows the possible comportment of SLNs in a polymeric matrix integrated into edible coating. In recent years, the usage of SLNs in the food industry has expanded greatly. SLNs provide practical delivery systems for lipophilic antioxidants, and nutraceutical and antimicrobial substances that may increase their stability, bioavailability, and dispersability in aqueous media [45]. Compounds like polyphenols, flavonoids, vitamins, minerals, oils (ω-3 fatty acids), carotenoids, lipophilic vitamins and phytosterols, among others, are all good choices as bioactive compounds that can be used to fortify food products and enhance their functionality [92]. Examples of applications of interest in food science are described in Table 2.

### 3.4. Incorporation of NLC Coatings

Food preservation, food safety, and the incorporation of colors, flavors, substances with characteristics and functionality for humans, antioxidants and antimicrobials, are some of the important applications of nanotechnology in the food industry [103]. One of the main advantages of nano-sized delivery systems in food is that they rarely affect the sensory attributes of the original product, so they can even be used in clear beverages [104].

Most applications of NLCs were introduced in pharmaceutical sciences, so there is little research on their applications in food-processing as a tool for delivering active substances through encapsulation and fortification [53,57]. Recent studies in this area focus on the development, characterization and in vitro analysis of systems that employ different natural lipids (solids and liquids), and on active ingredients obtained from natural sources, but not in the context of direct applications in a food matrix. Only a few authors have reported such NLC applications. For example, in 2015, Zhu et al. [105] prepared krill oil-loaded NLCs as a dietary supplement in a simulated beverage to study the feasibility of applying them in the beverage industry by measuring their stability during a storage period. Babazadeth et al. [57] reported the use of NLCs in beverages like milk and juices (orange and apple), and elaborated models for studying physical changes, such as the effects of pH and temperature on the stability and turbidity of rutin-loaded NLCs. Bagherpour et al. [54], meanwhile, studied the encapsulation of beta-sitosterol and its incorporation into butter to improve that food’s nutritional and antioxidant properties. Those research groups concluded that NLCs are a stable and suitable food-grade carrier for active substances that may be beneficial for future applications in the development of functional foods.

Table 3 presents the surfactants used in recent research. There, it is evident that the preferred surfactant for NLC production is Tween^®^ 80, a food-grade, hydrophilic non-ionic surfactant that is sometimes mixed with lecithin, a neutral high molecular weight surfactant which forms a bilayer that needs a cosurfactant to overcome the longer time needed to adsorb at the interface. The mixture of these two surfactants forms an internal (lecithin) and an external (Tween^®^ 80) membrane that decreases the zeta potential [45,58], thus increasing particle stability. Other common surfactants used are poloxamers and PEGylated surfactants.

In addition, nanotechnology offers great advantages in coating and packaging applications compared to conventional materials, by increasing food safety, production, and preservation time, while also enhancing shelf life by creating physical barriers. However, they present researchers with a challenge: the need to create edible delivery systems that are both safe and suitable for human consumption [103,106]. By improving targeting and controlled-release, nanostructures can increase the load solubility, dispersion, and bioavailability of active compounds.

Edible coatings have long been used to provide a physical barrier between the environment and food products, and so extend shelf life, but today the tendency is to include active food ingredients in coating formulations to improve and functionalize their properties. In this way, the quality and nutritional parameters of food products can be enhanced [107].

In this regard, nanotechnology will play an important role in future research on the development, characterization, and application of systems as coatings on foods destined for human consumption; NLCs have been shown to be a suitable choice for the application of nanotechnology in edible coatings, due to their high loading capacity, stability, and controlled release properties [22,47].

### 3.5. Inorganic Nanocomposites in Edible Coatings

**Figure 4:**
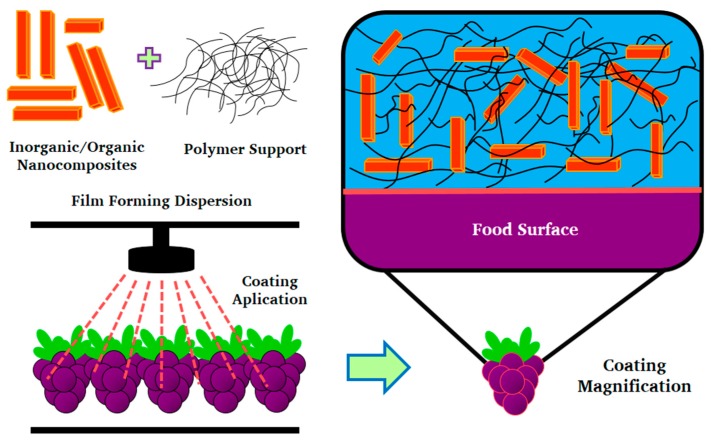
Inorganic nanocomposites incorporated in edible coating.

Inorganic compounds play an important role in the development of certain edible coatings, which require small proportions of such components. However, there is still much to explore, since there is an infinity of polysaccharides, proteins, and natural lipids that can be considered when developing edible coatings for different foods and, therefore, infinite possibilities will depend on the specific characteristics, origin, and composition of each food product. Table 4 shows some of the applications of nanocomposites include both organic and inorganic components, and can be explored for edible coatings. Figure 4 represents a schematic description to explain the way in which nanostructured systems interact in an edible coating.

### 3.6. Nanotubes and Nanofibers

**Figure 5:**
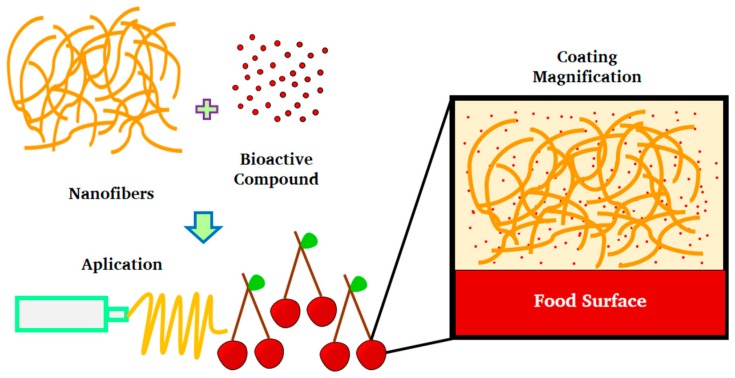
Nanofibers in edible coatings.

The electrospinning technique has made it possible to develop nanofibers that can be functionalized with different active substances that, preferably, have antimicrobial and antioxidant effect. This includes the use of biologically-active compounds, such as EOs from plant extracts often described as concentrated hydrophobic liquid-contained volatile substances. Figure 5 presents a coating formed by nanofibers embedded in a polymeric matrix, where release is performed by diffusion from nanofibers containing the active compound. On the other hand, nanocrystals are also an option for forming edible coatings. Nanocrystals modify the mechanical and barrier properties, due to their high crystallinity and negatively-charged structure, that allows greater affinity with the food surface. The incorporation of these nanosystems is also carried out in polymeric matrices (protein and polysaccharides), which can be functionalized with different EOs and/or plant extracts [115]. Table 5 summarizes the applications of these nanosystems in food preservation when employed as edible coatings.

## 4. Conclusions and Future Trends

Today, nanosystems represent an important area of food research, and the best candidates for the development of more efficient edible coatings with high potential in applications in food preservation. The main applications would be for fresh products, due to market preferences, since the elaboration of edible coatings with nanosystems allows the incorporation of antimicrobial and antioxidant ingredients. The ideal type of submicron system will depend on the characteristics of the food, the substance to be encapsulated, and the desired increase in shelf life. Preferably, the ingredients should be non-toxic and derived from natural sources, such that the functionalized nanosystem permits the controlled release of active substances with low solubility. Finally, the nanosystem chosen should contribute to maintain the functionality of the volatile compounds present in the essential oils and other plant extracts, in order for the latter to achieve the goal of protecting the food.

Future trends should include finding novel alternatives for controlling mechanical properties, gas transport, and thermal resistance, for the edible coating to adapt to environmental conditions by modifying its properties in relation to such factors as relative humidity and temperature. This requires selecting the appropriate type of nanosystem. To give but one example, the use of inorganic substances in nanocomposites will permit the development of systems with better control over the transport of O_2_, CO_2_, and water vapor.

However, despite the research emphasized here, it is clear that much more work is needed. In particular, we must understand the behavior of these materials after consumption, in order to develop safe nanosystems that can be used freely in commercial products.

## Figures and Tables

**Table 1:** Studies of the impact of nanoemulsions as edible coatings in different foods on shelf life, antioxidant capacity and antimicrobial and enzymatic inhibition.

Bioactive Substance	Functionality	Biopolymer Matrix	Food/Product	Findings
Carvacrol	Antimicrobial	-	Cabbage	The antimicrobial activity of a carvacrol nanoemulsion was proven from the results of inhibition of *E. coli* and *P. pastoris* growth in nutrient broth [73].
Carvacrol	Antimicrobial	Chitosan	Cucumber	The combination of pulsed light (12 J/cm^2^) with the edible coating (0.08% carvacrol) resulted in a strong synergistic effect, with E. coli reduction reaching >5 log cycles [ 74].
Cinnamaldehyde	Antimicrobial	Pectin (low and high methyl ester)	Edible films (in vitro)	The antimicrobial activity provided by cinnamaldehyde against food pathogens was remarkably improved by droplet size reduction due to increased surface area [ 68].
Cinnamaldehyde, garlic essential oil and α-tocopherol	Antioxidant	Gelatin Chitosan Sodium caseinate	Edible films (in vitro)	The best antioxidant activity and physical properties were evaluated for the film based on gelatin-sodium caseinate, indicating its potential use as an active edible coating and biodegradable packaging materia [ 75].
Clove bud and oregano essential oils	Antimicrobial and Shelf Life Extender	Methylcellulose	Sliced Bread	The films developed showed positive effects on yeast and mold counts compared to the commercial antifungal agent used [ 71].
Lemongrass essential oil	Antimicrobial	Sodium alginate	Fresh-cut apple	Nanoemulsion-based edible coatings presented higher *E. coli* inactivation and slower psychrophilic bacteria growth compared to conventional emulsions at the same concentration [76].
Lemongrass oil	Antimicrobial Antioxidant	Chitosan	Grape berry	The use of the nanoemulsion effectively reduced the initial growth of *S. typhimurium*, total aerobic mesophiles, yeasts and molds, and showed retention of antioxidant capacity [77].
Mandarin essential oil	Antimicrobial	Chitosan	Green beans	The combination of the bioactive coating and UV-C treatment reduced the *L. innocua* population and maintained the microbial load at a constant level during storage [78].
Oregano essential oil	Antimicrobial	Mandarin fiber	Low-fat cut cheese	High effectiveness on the inactivation of pathogens such as *Staphylococcus aureus*, and preserved the outward appearance of the cheese during the study period [79].
*Zataria multiflora Boiss.* essential oil	Inhibition of lipid oxidation	-	Rainbow trout fillets	The use of the nanoemulsion showed good-quality, protective features against lipid oxidation, including the peroxide value, free fatty acids and total volatile basic nitrogen during refrigerated storage [ 80].
α-tocopherol	Enzymatic activities and shelf life extender	Nopal mucilage	Fresh-cut apples	The coatings formed with the nanoemulsion had a significant inhibitory effect on PME and PPO activity, in contrast to conventional emulsions [ 46].

**Table 2:** Examples of SLN applications in the food industry.

Bioactive Compound	Matrix Lipid	Surfactant/Stabilizer(s)	Food Product	Application
-	Candeuba^®^S wax (carnauba wax and candelilla wax)	Poloxamer 407	Guava (*Psidium guajava* L.)	The potential use of SLNs in edible coatings could be applied easily to minimize the senescence of several products [ 93].
-	Candeuba^®^S wax (carnauba wax and candelilla wax)	Poloxamer 407	Edible Films (in vitro)	These findings suggest that SLN films have potential uses in preservation as nano-coatings for whole fruits and vegetables [ 94].
-	Candeuba^®^S wax (carnauba wax and candelilla wax)	Poloxamer 407	Guava (*Psidium guajava* L.)	The application of candeuba wax (SLN) helps to conservate the natural maturation process, but at a slower rate [ 95].
-	Glyceryl tristearate	Polyoxymethylene 20, sorbitan monolaurate, sucrose stearate and soy bean lecithin	Emulsion o/w	The presence of SLNs in emulsions led to increased emulsion stability as reflected by droplet size measurements and accelerated creaming experiments [ 96].
**Curcumin**	Glyceryl behenate	Poloxamer 188, soy lecithin and Polysorbate 80	In vitro	Increased the extremely low oral bioavailability of curcumin [ 97].
**Quercetin**	Glyceryl monostearate	Polysorbate 80, sorbitan monolaurate and lecithin	In vitro	Bioaccessibility increased significantly when incorporated into the SLN compared to free quercetin in its native form [ 98].
**Vitamin B2**	Fully hydrogenated canola oil	Polyethylene glycol (PEG) and sodium lauryl sulfate (SLS)	In vitro	It is possible to generate nano-scale solid lipid particles with a high content of a hydrophilic bioactive; however, further fine-tuning is needed [ 99].
**β-carotene**	Cocoa butter and/or hydrogenated palm oil	Polysorbate 80	In vitro	SLN may not be better than liquid lipid nanoparticles for encapsulating bioactive food ingredients [ 100].
**α-tocopherol**	Glyceryl behenate/soy lecithin	Soya lecithin, Poloxamer 188	In vitro	The stability of the SLN formulation was improved as well as the retention of α-tocopherol [ 101].
**Resveratrol**	Stearic acid	Poloxamer 188	In vitro	The lipid formulation produced a significant improvement in the oral bioavailability of resveratrol as compared to the intact suspension [ 102].

**Table 3:** Active compounds incorporated into NLCs for applications in food industry.

Active Compound/Functionality	Solid Lipid	Liquid Lipid (Oil)	Findings
Cardamom oil/Antimicrobial	Cocoa butter	Olive oil	NLCs had high entrapment efficiency (>90%), few changes were detected in the turbidity of systems after storage time with no significant aggregation and encapsulation was able to protect the antimicrobial activity of cardamom oil so in can be used as food supplements [ 56].
β-carotene/Pigment	Tristearin	Sunflower oil	β-carotene incorporation reduced the particles polydispersity and NLCs exhibited an improvement of β-carotene loading capacity compared with SLN. NLCs exhibited advantages over the SLN such as enhanced loading capacity and prevention of active expulsion [ 108].
Vitamin D/Antioxidant, calcium absorption	Glycerol monostearate	Oleic acid	In vitro digestion in simulated gastrointestinal fluids demonstrated their capability for controlled release because the NLCs were able to remain stable and protect the VD3 in simulated stomach fluid [ 109].
Pomegranate seed oil/Antioxidant	Beeswax, propolis wax	Glyceryl behenate	Lecithin, Tween 80Formulation variables had significant effects on physical properties of NLCs and presented excellent physical stability. The optimum formulations contained 10% oil and 6% surfactant [ 58].
Rutin/Nutraceutical, antimicrobial	Cacao butter	Oleic acid	NLCs with a rutin to lipid ratio of 10% were selected as an optimum formulation obtaining round shaped NLCs to fortify food samples as a method for designing new functional foods [ 57].
Betasitosterol/Anti-inflammatory, cholesterol reduction	Precirol	Miglyol	NLCs showed a high encapsulation efficiency (99.96%) and showed a good stability during three months’ storage period when incorporated in butter increasing acid and peroxide values as well as antioxidant properties [ 54].
Quercetin/Antioxidant	Glyceryl monostearate	Linseed oil	The addition of linseed oil improved the in vitro antioxidant activities of quercetin loaded NLCs exhibiting a sustained pattern. Lower lipid oxidation was found in quercetin and linseed oil co-loaded NLC compared with conventional linseed oil emulsion NLCs were stable for more than 3 months at 25 °C [ 59].
Resveratrol/Antioxidant	Lauric acid, stearic acid, cacao butter	Glycerol, oleic acid, miglyol, corn oil	The stability of different formulations was evaluated over 60 days of storage finding that the optimum formulation was reached by oil to solid lipid ratio of 15%, surfactant to emulsion ratio of 6% and storage at 20 °C for 30 min with sonication treatment [ 53].
Lycopene/Red color, antioxidant	Glycerol distearate, glycerol monostearate	Caprylic/capric triglyceride	Encapsulation efficiency of NLCs was significantly higher than SLNs. Glycerol monostearate containing nanoparticles showed phase separation after 30 days in 6 and 25 °C when incorporated in a beverage product. A sensory analysis indicated that nanoencapsulation could avoid the poor solubility and taste of lycopene [ 110].

**Table 4:** Nanocomposites in edible coatings to improve the mechanical properties and/or antioxidant and antimicrobial properties.

Nano-Inorganic Component	Functionality	Biopolymer Matrix	Food/Product	Findings
Nano-SiOx	Quality preservation, Shelf life extender	Soy protein isolate (SPI)	Apples	The preparation of edible a coating by ultrasonic processing and incorporation into an SPI matrix results in a decreased respiration rate, maintenance of firmness, and extension of shelf life [ 15].
Montmorillo-nite (MMT)	Antimicrobial psychotropic microorganism, (fungi and yeasts) Shelf life extender	Whitemouth croaker/ore-gano essential oil	Fresh-cut papaya, pear	Adding 15 g/L of montmorillonite at 80°C and essential oil of oregano decreased weight loss and maintained the quality of papaya; moreover, the edible coating helped slow microbial grow [ 111].
Montmorillo-nite (MMT)	Antifungal effect Increase storage time	Whey protein isolate (WPI)/calcium caseinate	Strawbe-rries	This edible coating contained 70% WPI, 0.5% potassium sorbate, 3.75% calcium caseinate and 0.375% MMT. It was effective in limiting mold growth during at least 12 days, and maintained the quality of the fresh coated strawberries [ 112].
TiO_2_	*E.coli., L. monocytogenes, S. aureus*	Cellulose nanofibers, WPI and rosemary essential oil	Lamb meat	The film coating with nano-TiO2 and rosemary reduced the growth of microorganisms more effectively and increased shelf life by 12–15 days [ 113].
Nano-ZnO_2_		Carboxymethyl cellulose (CMC)	Ready-to-eat pomegranate	Edible coatings with 0.2% ZnO_2_ were the most effective, decreasing yeast and mold growth at 6 and 12 days of storage, though the bacterial load increased after 12 days of storage. The combination of CMC with nano-ZnO_2_ helped maintain bioactive compounds in the pomegranate [ 62].
Silver nanoparticles (AgNPs)	*E. coli, S. aureus, Penicillium italicum*	*Fantasia japônica leaf extract*	Citrus fruit	AgNPs caused cell deformation, cytoplasmic leakage and cell death of *P. italicum*. AgNPs also showed significant activity on *E. coli and S. aureus* with beneficial effects for Citrus fruit preservation [15].
Silver nanoparticles (AgNPs)	Retention of volatile compounds	CMC/guar gum	Kinnow (*Citrus reticulata*)	Coating emulsion base and silver nanoparticles were mixed with CMC or guar gum at 1:1. The final concentration of Ag was 0.03 mg/L. The coating was applied to the fruit surface, finding that the ZnO_2_ coating helped maintain the volatile compounds of the products [ 114].

**Table 5:** Nanofibers and nanotubes utilized in edible coatings.

Nanotube/Nanofiber	Function	Biopolymer Matrix	Food	Conditions	Findings
Microfibrilla-ted Carrot (MC)	Improve mechani-cal properties	Starch	Carrot	Carrot MFC supensions were obtained after 20–40 pas-sages through the defibrillator	Reinforce mechanical properties of the edible coating and diminished permeability to water vapor, with which these possess good functionality and compatibility [116].
Avicel^®^ Cellulose NanoFibers (CNF)	Mechani-cal, glass transition (Tg)	Chitosan	Foods	0–20% CNF 0–30% glycerol	Finding that optimal concentrations to obtain a decrease in vitreous transition temperature were 15% of nanocellulose fibers and 18% of glycerol as plasticizer [115].
Cellulose NanoFibers (CNF)	Gas barrier and mechani-cal resistance	Fish Gelatin (FG) Palmitic acid	Foods	2% CFN and 6% FG	It was found that the use of CFN as reinforcement for edible coatings contributed to improving the properties of the water- vapor barrier and mechanical strength [117].
Zein nanofibers	Encap-sulated curcu-min, anti-microbial agent	curcumin	Apples	Electrospun zein (2.5–5%)	The surface was inoculated with *Botrytis cinereal* and *Penicillium expasum*; then, apples were coated by electro-spinning with zein nanotubes and storage for 15 days, revealing the inhibi-tion of microbial growth increase in the shelf life of apples [10].
